# Cultural adaptation of a peer-led lifestyle intervention program for diabetes prevention in India: the Kerala diabetes prevention program (K-DPP)

**DOI:** 10.1186/s12889-017-4986-0

**Published:** 2018-01-04

**Authors:** Elezebeth Mathews, Emma Thomas, Pilvikki Absetz, Fabrizio D’Esposito, Zahra Aziz, Sajitha Balachandran, Meena Daivadanam, Kavumpurathu Raman Thankappan, Brian Oldenburg

**Affiliations:** 1 Achutha Menon Centre for Health Science Studies, Sree Chitra Tirunal Institute for Medical Sciences and Technology, Thiruvananthapuram, Kerala India; 2 Department of Public Health and Community Medicine, Central University of Kerala, Kasaragod, Kerala, India; 3 Melbourne School of Population and Global Health, University of Melbourne, Melbourne, VIC Australia; 4 Department of Public Health and Clinical Nutrition, University of Eastern Finland, Kuopio, Finland; 5 School of Health Sciences, University of Tampere, Tampere, Finland; 6 Collaborative Care Systems Finland, Helsinki, Finland; 7 Department of Food Nutrition and Dietetics, Uppsala Univeristy, Uppsala, Sweden; 8 Department of Public Health Sciences, Karolinska Institutet, Stockholm, Sweden

**Keywords:** Cultural adaptation, Diabetes prevention, Type 2 diabetes mellitus (T2DM), Low and middle income countries (LMICs), Community-based, Peer support, Lifestyle intervention, Implementation

## Abstract

**Background:**

Type 2 diabetes mellitus (T2DM) is now one of the leading causes of disease-related deaths globally. India has the world’s second largest number of individuals living with diabetes. Lifestyle change has been proven to be an effective means by which to reduce risk of T2DM and a number of “real world” diabetes prevention trials have been undertaken in high income countries. However, systematic efforts to adapt such interventions for T2DM prevention in low- and middle-income countries have been very limited to date. This research-to-action gap is now widely recognised as a major challenge to the prevention and control of diabetes. Reducing the gap is associated with reductions in morbidity and mortality and reduced health care costs. The aim of this article is to describe the adaptation, development and refinement of diabetes prevention programs from the USA, Finland and Australia to the State of Kerala, India.

**Methods:**

The Kerala Diabetes Prevention Program (K-DPP) was adapted to Kerala, India from evidence-based lifestyle interventions implemented in high income countries, namely, Finland, United States and Australia. The adaptation process was undertaken in five phases: 1) needs assessment; 2) formulation of program objectives; 3) program adaptation and development; 4) piloting of the program and its delivery; and 5) program refinement and active implementation.

**Results:**

The resulting program, K-DPP, includes four key components: 1) a group-based peer support program for participants; 2) a peer-leader training and support program for lay people to lead the groups; 3) resource materials; and 4) strategies to stimulate broader community engagement. The systematic approach to adaptation was underpinned by evidence-based behavior change techniques.

**Conclusion:**

K-DPP is the first well evaluated community-based, peer-led diabetes prevention program in India. Future refinement and utilization of this approach will promote translation of K-DPP to other contexts and population groups within India as well as other low- and middle-income countries. This same approach could also be applied more broadly to enable the translation of effective non-communicable disease prevention programs developed in high-income settings to create context-specific evidence in rapidly developing low- and middle-income countries.

**Trial registration:**

Australia and New Zealand Clinical Trials Registry: ACTRN12611000262909. Registered 10 March 2011.

## Background

Type 2 diabetes mellitus (T2DM) is now one of the leading causes of disease-related deaths globally [1]. India has the second-largest number of individuals with T2DM in the world (currently, estimated to be around 70 million); and there are a similar number of individuals at high risk of progressing to diabetes [2]. Indian regional and national studies estimate that the condition affects between 9% and 20% of the adult population [3–5] with India’s southernmost state of Kerala, having the highest prevalence of T2DM (at least 20%) [6, 7].

Several large efficacy trials including the Diabetes Prevention Study in Finland (Fin-DPS) [8], the United States Diabetes Prevention Program (US DPP) [9], as well as diabetes prevention trials in Japan [10], China [11] and India [12] have demonstrated that lifestyle change can reduce T2DM incidence by up to 60% in high-risk populations. Following the success of these efficacy trials, efforts have been made to replicate these findings in more real world contexts. Absetz, Oldenburg and their colleagues used the Fin-DPS as a benchmark for the Good Ageing in Lahti (GOAL) Region Lifestyle Implementation Trial in Finland [13, 14], and the Greater Green Triangle Diabetes Prevention Program (GTT DPP) adapted and tested the GOAL model in Australia [15]. These studies have now been followed by a number of translational studies based on either the Fin-DPS or the US DPP model in other high-income countries (HICs) such as the United States [16–18], United Kingdom [19, 20], Netherlands [21], Europe [22–24], Australia [25] and Japan [10, 26]. In the case of the US DPP, the Centers for Disease Control and Prevention (CDC) have now developed a curriculum to ensure adapted programs meet requirements for recognition. In Finland, the GOAL program has been disseminated in several regions across the country with a standard protocol for implementation, including program materials, and facilitator training and certification. Such protocols are paving the way forward for future adaption of diabetes prevention programs, whilst ensuring fidelity of the original evidence-based intervention is maintained.

To date, cultural adaptation of T2DM prevention programs has mainly occurred with ethnic groups or indigenous population within HICs and has included settings such as churches [27], health centres and community centres [17, 28–37]. Efforts to adapt programs and models of delivery for T2DM prevention in low- and middle-income countries (LMICs) have been very limited. Indeed, a recent systematic review of 38 well evaluated real world diabetes prevention studies reported no such programs from LMICs [38]. This presents a large evidence gap given LMICs differ substantially in terms of health systems, resources, culture, and lifestyle risk factors. Therefore, context-specific evidence is required and well-warranted given reducing the evidence-to-action gap is associated with reductions in morbidity and mortality and reduced healthcare costs [39–41]. There is now an urgent need to develop models and approaches to reduce the risk of developing T2DM in LMICs. This is particularly pertinent in a country like India where the disease burden is large and rapidly growing. In order to create impact at the population level, such programs require approaches that are community-wide and scalable.

To address this critically important evidence-practice gap, our team has developed the Kerala Diabetes Prevention Program (K-DPP), a culturally tailored, community-based and peer-led diabetes prevention intervention for individuals at high risk of developing T2DM in the state of Kerala, India. This paper describes the development and piloting of the program and we discuss the learnings so far and the implications for future program adaptation elsewhere. Although screening and recruitment are important parts of diabetes prevention programs, it is outside the scope of this paper to describe the process undertaken in K-DPP, however, details have been previously published [42]. The description of the needs assessment and the intervention protocol for K-DPP have also been published earlier [43, 44].

## Methods

**Fig. 1:**
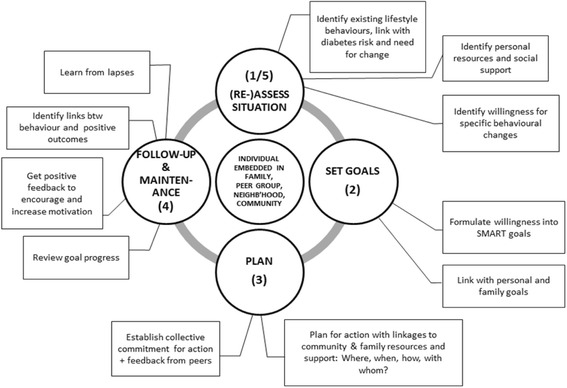
Overview of the Kerala Diabetes Prevention Program lifestyle change model

Due to the lack of evidence-based diabetes prevention programs in India and other LMICs [38], the K-DPP program was adapted to Kerala, India from the GOAL Lifestyle Implementation Trial in Finland [13, 14], the US DPP [9, 45] and the GGT DPP in Australia [15]. The adaptation process was undertaken in five phases: 1) needs assessment; 2) formulation of program objectives; 3) program adaptation and development; 4) piloting of the program and its delivery; and 5) program refinement and active implementation. We used Intervention Mapping to guide these five phases [46]. The development and cultural adaptation of the program focused explicitly on maintenance of behavior (see Fig. 1) and the potential for future scalability and sustainability. The research was approved by the Institutional Ethics Committee of the Sree Chitra Tirunal Institute for Medical Sciences and Technology, Thiruvananthapuram, India (SCT/IEC-333/May 2011), and by the Human Research Ethics Committee of Monash University, Australia (CF11/0457-2,011,000,194) and the University of Melbourne, Australia (1441736). The trial was registered on the Australia and New Zealand Clinical Trials Registry: ACTRN12611000262909.

### PHASE 1: Needs assessment

The needs assessment phase involved the triangulation of: available evidence from policy and program documents relevant to diabetes prevention in Kerala and India; empirical studies on the prevalence and control of diabetes in India; and lay perceptions of T2DM from focus groups held in communities where the program was to be delivered. The complete detail of the needs assessment has been previously published, [43] however, in brief, a literature review of relevant Indian Government sites was undertaken to search for policy documents related to national and state capacity, legislation, programmes and guidelines for non-communicable disease (NCD) prevention and corroborated with key Government officials in Kerala for accuracy. A literature review was also undertaken on empirical studies related to diet, physical activity, and tobacco use in relation to NCDs in India. Further a sub-group of individuals with pre-diabetes identified through an earlier community-based survey in Trivandrum were contacted and invited to take part in a focus group discussion (FGD). Four main areas were discussed during the groups including: 1) participants’ understanding of diabetes and interest to know more; 2) health information sources and access to them; 3) participants’ motivation to participate in a community-based diabetes prevention program; and 4) how such a program should best be delivered [43]. Main themes from the FGDs were identified through content analysis.

### PHASE 2: Formulation of program objectives

Due to the paucity of intervention studies underpinned by socio-behavioral theories in the Indian context, there existed very limited evidence on the application of behavior change theories and determinants of target behaviors. As such, the Health Action Process Approach model [47] was utilised to identify evidence-based determinants including: low outcome expectations, low risk perception, low self-efficacy, and lack of social support for healthy lifestyle. The objectives and the determinants were translated into ‘personal learning objectives’ for the participants such as ‘increasing awareness of T2DM risk factors’ in addition to ‘environmental change objectives’ such as ‘enhance peer support for behaviour change’. Further, the program objectives for K-DPP were heavily informed by the needs assessment, particularly the findings from the focus groups. Using the Intervention Mapping approach [46], the themes which emerged from the needs assessment and literature were then translated into target behaviours with corresponding determinants of behavior and environmental conditions.

### PHASE 3: Program adaptation and development

During Phase 3, the program objectives along with the findings from the needs assessment were used to inform the development of the K-DPP intervention model and its delivery appropriate to the Keralan context. The suggested behavior change techniques were mapped onto Michie et al.’s Behavior Change Technique (BCT) Taxonomy v1 [48]. The BCT is a hierarchically grouped, consensus-based taxonomy of 93 techniques and aims to improve the reporting of behavioral interventions. To address the key determinants in the target communities, several behavior change techniques were identified along with feasible and culturally acceptable strategies to enhance program engagement and implementation which emerged from the FGDs.

### PHASE 4: Piloting the program and its delivery

The program was piloted in one of the communities randomly selected from the trial sampling frame. Participants were selected using the Indian Diabetes Risk Score, the details of which have previously been described [44]. The pilot program included: an inaugural session where participants were briefed about the program and provided with the participant resource material; peer-leader selection and training; a diabetes education session; and the first four small group sessions. Participants and their family members were encouraged to attend all aspects of the pilot program. Small group sessions were conducted at a school facility on Sundays, as this was the only day when both men and women could attend. The aim of this pilot program was to: assess recruitment of participants, delivery of the intervention components, and participant retention. Additionally, the program materials were piloted to assess their appropriateness, comprehensiveness, and responsiveness to gender and cultural sensitivities. After the completion of the pilot program, participants provided feedback to members of the study team through an informal discussion on the program delivery, content of the resource materials and strategies for participant retention.

### PHASE 5: Refinement and active implementation

The findings from the four developmental phases then informed program implementation as part of a cluster randomized control trial. Participants (intervention arm, *n* = 500; control arm, *n* = 507) were recruited directly from the community through home visits. Participants were at high-risk of diabetes (Indian Diabetes Risk Score ≥ 60 and were without T2DM on oral glucose tolerance test), aged 30-60 years (mean age 46.0 ± 7.5 years) and 47.2% were women [42]. During the implementation, the focus broadened from fostering engagement and participation in the K-DPP peer groups to preparation, adoption and maintenance of behaviour changes by individuals and their families; and finally to community empowerment.

## Results

### PHASE 1: Needs assessment

The needs assessment revealed a paucity of policy and research on NCDs in Kerala and India in spite of the large burden of NCDs across the state. The available research suggested that K-DPP was to be implemented in a setting where there was limited attention to the prevention of NCDs and no widely implemented programs related to diabetes prevention.

Three FGDs were held, each with six participants (*n* = 18; age range 33-64 years). All participants had pre-diabetes (fasting blood glucose 110-125 mg/dl) and came from the Thiruvananthapuram District. Content analysis of the FGDs revealed: a general interest to know more about diabetes and its prevention; the commonplace and somewhat ‘normalized’ nature of lifestyle risk factors such as unhealthy diet and physical inactivity; a lack of awareness of T2DM risk; a limited understanding of measures to prevent T2DM; and low self-efficacy regarding the ability to make and sustain lifestyle changes [43].

### PHASE 2: Formulation of program objectives

The target health-related behaviors, and hence the program objectives for lifestyle change, were similar to diabetes prevention studies in HICs. Table 1 shows the identified program objectives as well as the modifiable behavioural and environmental determinants for the program objectives. These included: moderate weight loss, increased intake of fibre, reduced total and saturated fat and increased physical activity. Other important lifestyle targets that emerged through the needs assessment, included: a reduction in carbohydrates with high glycaemic index such as refined rice and sugar containing foods and beverages; improved sleep; reduction of smoking and chewing tobacco; and the reduction of alcohol (among males).

**Table 1:** Kerala Diabetes Prevention Program objectives, theory-based methods and practical strategies

Program Objectives	Participant learning and environmental change objectives	Theory- and evidence-based determinants as per the Health Action Process Approach [47]	Behavior change techniques as per Michie et al.’s Taxonomy v1 [48] (BCT number)	Feasible and culturally acceptable strategies to enhance engagement and implementation
1. Increase the consumption of fruit, vegetables and fibre 2. Reduce intake of carbohydrates with high glycaemic index and total and saturated fats 3. Increase physical activity 4. Reduce tobacco use with emphasis on chewing tobacco 5. Reduce alcohol consumption, particularly among men 6. Set realistic goals and associated targets for weight loss and other lifestyle risks 7. Improve sleep	Participant learning objective • Increase awareness of the risk factors of T2DM • Improve risk perception on T2DM • Improve self-efficacy in making lifestyle changes Environmental change objective • Enhance peer support for behavior change • Enhance household / family support for behavior change • Enhance neighborhood and community support • Facilitate opportunities for healthy life style with collaboration at group-community level.	• Outcome expectations • Risk perception • Self-efficacy • Action planning • Coping planning	• Goal setting (behavior) (BCT #1.1), action planning (BCT #1.4) and review of behaviour goal(s) (BCT #1.7) e.g. participants are assisted to set realistic behavioral goals and prompted to detail a plan of how they will achieve it. The goals are reviewed within the sessions. • Instruction on how to perform a behaviour (BCT #4.1) e.g. experts advised and up-skilled participants in yoga classes and kitchen garden development • Information about health consequences (BCT #5.1) e.g. information is provided in the DPES sessions and small group sessions on diabetes and potential complications • Problem solving/coping planning (BCT #1.2) e.g. barriers to physical activity and healthy eating are discussed and planned for throughout the small group sessions • Social support (practical) (BCT #3.2), social support (general) (BCT #3.1), and social support (emotional) (BCT #3.3) e.g. inclusion of family members and peer-based intervention is designed to enhance social support	Individual-level • Educational sessions that focus on ‘modifiable’ determinants of risk on diabetes • Provide information on the risk factors of T2DM • Sessions scheduled in local neighborhoods (e.g. a reading room or *anganwadi*) according to work, family and other cultural needs of participants • Inclusion of strategies to attract more male participation Interpersonal-level • Group-based delivery/ peer-support • Inclusion of family members in the K-DPP sessions • Provide information on the dietary and physical activity targets for individuals as well as family members • Enabling ongoing peer and social support, with family members and friends of participants • Kitchen gardening training and seeds • Forming of walking groups • Yoga training sessions Community-level • Community mobilization activities • Forming partnerships with community stakeholders and organizations • Clearing of walking paths with peer group and community members

### PHASE 3: Program adaptation and development

#### Behavior change techniques

As per Table 1, the resulting behavior change techniques included: goal setting and action planning, information about health consequences, problem solving/coping planning and social support. The FGDs especially highlighted the important role that families and cultural norms play in decision-making related to lifestyle choices in India, which underpinned the importance of developing a more collectivistic approach to behavior change interventions than was adopted in previous studies. Peer support, defined as “emotional, social, and practical assistance provided by non-professionals to help people sustain health behaviors” [49] was also identified as a potential technique to enhance behaviour change.

#### Strategies to enhance engagement

Various strategies were suggested to enhance engagement with the intervention, including the involvement of family members and community mobilization activities (see Table 1). These were corroborated from other studies in India [50–56]. In order to link with the needs of the broader community, each peer support group was encouraged to identify prevention activities that might engage other individuals in their community. It was proposed, for example, that they consider community activities such as walking groups, kitchen gardens and yoga groups. Through empowering the community to participate in lifestyle changes, favourable social norms and enabling local environments would more likely be created. Identification and empowerment of key stakeholders in the community such as leaders, citizens, organizations, and volunteers was recognized as a fundamental success factor for this process.

#### Intervention model and delivery

**Fig. 2:**
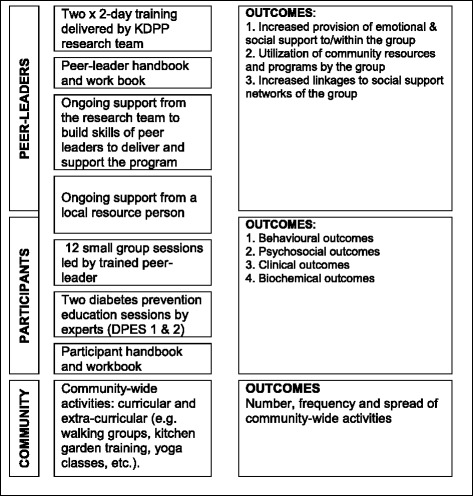
Kerala Diabetes Prevention Program components

To deliver the elements outlined above, a program was formulated with community-based peer group meetings as the core delivery strategy, centrally organized Diabetes Prevention Education Sessions (DPES) to share knowledge more broadly, as well as additional community activities to assist in achieving program goals and maintenance (see Fig. 2). Forming partnerships with public sector health care and other community-based organizations was identified as a key factor for successful implementation of other widely implemented diabetes prevention programs in HICs. However, in Kerala, the public sector health care only caters to a minority of citizens; indeed, even among the socioeconomically disadvantaged, the majority of individuals choose to seek private care [56]. Therefore, rather than being implemented and delivered directly through the health care system, it was decided that K-DPP should link more directly with community stakeholders such as leaders of local self-government bodies, called the Panchayats. Each Panchayat nominated a local resource person, mostly Accredited Social Health Activists (ASHAs) to support implementation. The tasks of the local resource person included: practical organization of the small group meetings, reminding and following up with participants, advocating the program, helping to set up extracurricular activities, and acting as a liaison between the group members and other community based organisations for efficient program uptake. The local resource person also attended small-group sessions as an observer and supporter of the peer leader, whenever possible. Hence, the program was designed to be delivered by lay peer-leaders and local resource persons, instead of health personnel.

### PHASE 4: Piloting the program and its delivery

A total of 26 participants (M = 7; F = 19) aged 31- 60 years were enrolled in two separate groups to pilot the program. The mean attendance rate of participants for the four small group sessions was 46.1% (*n* = 12 participants). Male participants had lower attendance rates (2/7) compared to females (10/19). A total of 28 family members also attended the pilot sessions.

The key challenges identified during the pilot phase were related to the perceived relevance of the program, the readability of resource materials, and attendance of male participants. As per Table 2, strategies were developed to address each of these challenges and the program was then modified accordingly.

**Table 2:** Major findings from the pilot phase and modifications made to the Kerala Diabetes Prevention Program

Identified challenge	Strategies adopted	Modifications made
Low education level of the participants. The majority of the participants (*n* = 18) had no formal education, with the highest level of education being 11 years of schooling.	Simplify intervention materials to assist understanding of individuals with lower literacy levels.	Intervention materials were modified with additional pictures to support understanding of text-based information. Additional group-based activities were planned to be incorporated into the sessions to facilitate story-telling and oral language based learning.
Low participation level of male participants.	Recruit male peer-leaders that can encourage male participants to attend. Ensure sessions are run during convenient times for working males.	Male peer-leaders were recruited in addition to the female peer-leaders. Sessions were organised during the evening and on weekends to enhance male participation.
Perceived relevance of T2DM prevention, with priority given to control and management of T2DM	A strong link between prevention and disease management needed to be established to make the program relevant for the participants. Program content (intervention materials and sessions) needed to be modified to sensitize participants on the need for diabetes prevention amongst themselves and their families and to include information on diabetes management. More community awareness on prevention programs was required.	An additional educational session, Diabetes Prevention Education Session (DPES 1), was incorporated into the program. DPES 1 provided an introduction to understanding Type 2 diabetes and its risk factors. This session stressed the similarity of strategies for primary and secondary prevention, and addressed misconceptions and role of lifestyle modification. The original diabetes education session became a sequel to DPES 1. This session, DPES 2, focused on the modifiable risk factors for diabetes prevention. The session took a deeper view on the specifics of healthy lifestyle behaviors, diet, physical inactivity, tobacco and importance of sleep. We also included “Diabetes Management” as an additional topic into the small group sessions to link diabetes management with prevention strategies, and thereby to increase perceived relevance of the program among participants.

#### Perceived relevance of T2DM prevention

Participants and their families were very interested in learning about T2DM, as reflected by the very high attendance in the DPES with 18 participants and 69 family members. However, many participants had family members or neighbors living with T2DM and were initially more interested in management of diabetes rather than its prevention. This was expressed in concerns such as *“How do we have time for prevention when we have to take care of our elderly parents who already have the disease?”* In order to enhance the relevance of diabetes prevention, it became very clear that the program needed to emphasize the similarity of lifestyle change strategies for prevention and management of diabetes, and show that by engaging in K-DPP, the participants could achieve the benefits of improving the management of diabetes in those living with the disease in the household, diabetes prevention and easing the burden of disease on their children as the future caregivers of aging parents and grandparents.

#### Readability of resource materials

Although the official literacy level in Kerala is over 90% [57], a high proportion of participants could not read and write in practice, as most of them had not used these skills for many years. This posed a limitation for the effective use of the intervention materials, which were consequently modified to contain more pictures and less text.

#### Attendance of male participants

Participation of men was lower than women during the pilot program. To enhance participation, both male and female peer-leaders were recruited and sessions were delivered in the evenings or on weekends to facilitate working males to attend. Peer-leaders were also asked to support participation by contacting the participants and discussing unattended sessions, and encouraging participation in further sessions.

### PHASE 5: Refinement and active implementation

The program was refined to facilitate engagement, adoption, maintenance, and community empowerment across the period of program implementation (Table 3). Systems to enhance support were directly built in to the program (e.g. pre- and post-session telephone contact, promoting linkages with community organizations) and activities were planned to directly engage peer leaders, participants, families, and their communities (see Table 3).

**Table 3:** Kerala Diabetes Prevention Program Components and focal areas of influence on different stages of intervention

	Engagement (0-2 months)	Preparation and adoption of changes (3-5 months)	Adoption and maintenance of changes (6-12 months)	Community empowerment (>9 months)
Overall objective	• Increasing willingness to participate • Rapport building • Establishing personal relevance • Increasing awareness of T2DM prevention and K-DPP	• Increasing personal relevance • Preparing for changes	• Increasing self-efficacy • Making and assessing changes on personal and family level	• Assessing and sustaining changes on personal and family level • Supporting community change
K-DPP Components	• Recruitment of LRPs • Small group sessions (1-2) • DPES 1 • Peer-leaders selection and training	• Small group sessions 3-5 • Pre- and post-session telephone contact with PLs and LRPs • DPES II • PLs training	• Small group sessions 6-12 • Pre- and post-session telephone contact with PLs and LRPs • Extra-curricular activities (yoga training, kitchen garden cultivation, etc.)Workshops for PL and LRP and support for planning extra-curricular activities in the community (E.g. healthy snack preparation, sports, painting competition on behavior change themes)	• Linkage with other services for health care and promotion • Linkage with other community organizations
Peer Leader (PL)	• Selection, • Commitment	• PL leader skill-building and support for self-efficacy • Benefits of being a PL	• Supporting PL self-efficacy and perception of benefits • Enabling and promoting peer support among peer-leaders	• Supporting peer-leader self-efficacy, autonomy and perception of benefits. • Promoting linkages with community organisations.
Participants (and family)	• Recruitment • Retention: participatory methods and benefits from participation (for participant and family)	• Building peer support and self-efficacy in behavior change in participant and family	• Promoting maintenance of peer support and behavior change • Supporting participants in becoming change agents in their families	• Promoting maintenance of peer support and behavior change in participant and family • Supporting participants in becoming change agents in their community
Community	• Increasing community awareness of K-DPP • Encouraging community support of K-DPP	• Encouraging community support of K-DPP	• How can K-DPP groups support health in their communities: extra-curricular activities and linkages with community organizations	• Support for community rollout

#### Kerala diabetes prevention program components

The resulting program, K-DPP, includes four key components summarized in Fig. 2: 1) a group-based peer support program for participants; 2) a peer-leader training program for lay people to lead the groups; 3) resource materials; and 4) strategies to stimulate broader community engagement.

1. ***A group-based peer support program for participants***

The K-DPP curricular activities include two diabetes education sessions (DPES 1 and DPES 2) and twelve peer-led small group sessions.

*Diabetes Education Sessions (DPES 1 and DPES 2)* were delivered by local healthcare experts to the program participants from 2 to 3 neighborhoods within close proximity. The aims of the sessions were to increase knowledge of T2DM and its risk factors, targets for behavior change and to highlight the benefits of participating in the small group sessions. DPES 1 provided basic information on T2DM and its management. DPES 2 focused on the rationale behind and means for modifying the risk factors to prevent T2DM. As in the pilot, participants were encouraged to bring their family members along to both DPES 1 and DPES 2.

*Each K-DPP group* comprised 10-20 people recruited from the same community. The small group sessions were delivered at locations convenient for participants, such as community centres, local reading rooms, and schools. The first four sessions took place fortnightly, and subsequent sessions were conducted monthly over the duration of ten months. Each session lasted for 60 to 90 min. The program started with an inaugural small group session, where participants were briefed about the program and provided with the participant resource material. The participants from each group selected two peer-leaders (a male and a female), from within the group. Subsequent small group sessions covered six topics: a) healthy diet (portion size, identifying cooking substitutions to reduce fat content in food); b) approaches to increase physical activity (finding enjoyable activities for individuals and building these into daily routines, avoiding injuries); c) weight loss (weight and waist measurement and ensuring intake of sufficient variety of foods while reducing calories); d) tobacco control and cessation; e) alcohol consumption reduction; and f) adequate sleep. Various strategies were employed to enhance participants’ knowledge on how to reduce their diabetes risk, such as through information provision, storytelling and problem solving. Sessions were planned to be flexible in style and management. The local resource person informed the participants of the time and venue of the sessions either by home visit or telephone call, and followed up attendance. Participants were encouraged to send another family member to any small group sessions where they were unable to attend themselves. This provided an opportunity for participants to understand what occurred during the sessions they may have missed and to spread the knowledge of the program among family members. If a participant missed two or more consecutive sessions, telephone calls were made to the participant by the K-DPP research team, followed by house visits by the local resource persons. Table 3 summarizes the activities conducted during the implementation of KDPP in relation to the participants and their families.

2. ***Peer-leader training program and ongoing support***

Peer-leaders, both male and female, were nominated from each group based on their level of education, willingness to lead the group, social credibility and acceptance by their group members. Peer-leaders underwent two training blocks of two days duration each. The first training block focused on knowledge and skill building in relation to diabetes prevention and group facilitation. In the second training block, taking place after the fifth small group session, experiences in conducting sessions were shared, and strengths as well as need for support from the K-DPP team and ways to improve the conduct of the sessions were identified. Each of the training blocks were attended by peer-leaders from seven to eight clusters, with at least one peer-leader from each cluster (average number per training block = 12 peer leaders). Peer-leader training was important for the initial building of knowledge and skills, but also provided a platform for sharing and support between the peer-leaders and helped to sustain motivation and confidence. The K-DPP intervention team provided on-going support to peer-leaders via telephone before and after each small group session. Additionally, peer leaders participated in a “peer leader’s follow up session” to brief on the activities conducted in their locality involving potential community stakeholders for knowledge dissemination and community partnership for healthy lifestyle behaviors. These stakeholders included: members from Kudumbashree State Mission (a community-based, poverty reduction project of the Government of Kerala); residents’ associations; arts and sports club members and organizers; and members from religious organizations. Such forums enabled the peer leaders to have varied perspective of integrating the program to the existing community networks.

3. ***Resource materials***

Each participant received a Participant Handbook and a Participant Workbook in the local language (Malayalam). The Participant Handbook contained information about T2DM, its risk factors, target behaviors and strategies to assist in behavior change, which the peer-leader or the participants could refer to during the sessions. The Participant Workbook includes tools for self-monitoring of behavior; goal settings for diet, physical activity, tobacco and alcohol use; and goal review. In addition, peer-leaders received a Peer-Leader Handbook, which expanded upon the Participant Handbook and Workbook and has hands-on instructions on how to facilitate the activities guiding the work during each session.

4. ***Strategies for community engagement***

The program implementation involved strong collaboration and engagement with the Panchayats (leaders of local self-government bodies). Each Panchayat nominated a local resource person, mostly ASHAs from the State public health services to support implementation. The local resource person was the first point of contact for each community and their role remained strong throughout the program. The involvement of family and community members was also encouraged to support the participants’ lifestyle changes through creating favourable social norms. Engagement of community members was enhanced through community-based activities held in the local neighborhoods. These included events to be conducted separately from the regular group sessions to assist the group members to make their behavioral goals practical such as walking groups, yoga clubs, and kitchen garden training to promote the cultivation and consumption of vegetables. Additionally, the role and reputation of the Indian institution behind the K-DPP project, the Sree Chitra Tirunal Institute for Medical Science and Technology (SCTIMST), was seen as an important contributing factor to program engagement. SCTIMST is an institute of national importance established by an Act of the Indian parliament in 1980 and has a positive reputation in the Kerala society. The Institute has a longstanding history of delivering patient care of high quality, technology development of industrial significance and health research studies of social relevance. The Achutha Menon Centre for Health Science Studies, the public health wing of SCTIMST is recognized as a centre of excellence for public health.

## Discussion

While 80% of NCDs occur in LMICs [58], the majority of the research evidence on how to tackle NCDs still derives primarily from HICs. Significant effort has gone into translating diabetes prevention studies to diverse populations within HICs such as ethnic or indigenous populations [17, 28]. The most widely translated and evaluated diabetes prevention program is the US DPP. A 2015 systematic review found 44 studies evaluating the US DPP across a range of settings and populations including various minority groups and populations with low socio-economic status [59]. The Centers for Disease Control and Prevention (CDC) have now developed a curriculum to ensure adapted US DPP programs meet requirements for recognition including complying with the Standards and Operating Procedures [60] and as well as completing a capacity assessment to ensure organizations are able to start and maintain programs successfully. In contrast to efforts to preventing diabetes in HIC, adaptation of programs to LMICs has been quite limited to date. Extending evidence-based interventions to LMICs and creating new context-specific knowledge is critically important. The WHO Global Action Plan for Prevention and Control of Non-communicable Diseases [61] calls for translational research to enhance the knowledge base for national, regional and global action on NCDs in LMICs. K-DPP provides one example of how this process can commence.

This study highlights the alignment of implementation science with cultural adaptation, both of which ultimately aim to translate and deliver interventions to improve the use and impact of evidence-based practices in new contexts. Cabassa et al. [62] states that using an approach to cultural adaptation that is systematic and which is aligned with implementation science can help narrow the gap between evidence and practice in four important ways: (1) by deepening the explicit attention and use of culture making interventions more responsive to the needs and preferences of diverse populations, (2) by specifying what to adapt in order to achieve optimal balance between adaptation and fidelity; (3) by expanding the attention to contextual factors that impact how adapted interventions are ultimately used and sustained in real-world settings, and (4) by specifying when in the implementation process adaptations may be most needed to enhance the adoption and sustainability of evidence-based practices.

As K-DPP originates from programs in Finland [13, 14], the USA [45], and Australia [15] translation to the Indian-context required significant adaptations in order to align with the target population, the culture and also the health system with its multiple actors (mainly for-profit) focused primarily on curative services. Adapting a policy or intervention to the context in which it will be delivered is a delicate balancing act: on the one hand adaptation is crucial to ensure relevance to the local context, improve feasibility, increase local pertinence and adoption, encourage fidelity, foster sustainability and maximize effectiveness; on the other hand, one has to be careful not to modify the policy or intervention so much that fidelity to some of the core components of the policy or intervention is lost and effectiveness is threatened [62].

The short history of NCD prevention and the paucity of relevant research in India made it crucial for our research group to conduct a comprehensive needs assessment and a more careful, phased program planning and adaptation process than had typically been the case in implementation studies in the HIC’s. The needs assessment showed a high prevalence of the behavioral risk factors and NCDs in the State of Kerala, with lack of services and mechanisms within the health system to curb the rising rates of disease [43]. This phase highlighted the importance of carrying out a thorough assessment of the context within which an intervention is to be implemented. Indeed, differences in culture, language, age and socioeconomic status of the target population have been shown to influence successful implementation of an intervention either positively or negatively [63]. Within the Kerala context, this phase revealed the importance of a collectivist approach to behavior change techniques with decision-making at the family level. The need for such an approach has been further supported by more in-depth studies on health behaviors, particularly dietary behavior change in relation to NCDs in this population [64]. The paucity of evidence on community-based life style interventions in India made it imperative to define context-specific objectives and strategies for personal and environmental change.

The pilot study provided insights into the challenges of implementation and enabled suggestions for feasible and effective ways of adapting the program to reflect the community needs. First and foremost, a strong link between prevention and disease management needed to be established to make the program relevant for the participants. Additionally, we encountered problems with male participation. Despite actions such as incorporating male peer-leaders and adjusting session timing to fit working schedules, attendance by male participants remained lower than female’s throughout the program. This challenge may be explained in part by a perception that health related activities are predominately linked with women. Until recently, the only health service delivered at a local level was maternal and child health with women being the major focus [65]. Strategies to engage males in health prevention programs require much greater attention in future research.

The implementation of the program highlighted many lessons that are likely transferable to other community-based prevention projects. Firstly, engagement of participants, peer-leaders and local resource persons was crucial. The high reputation of the implementing institute (SCTIMST) was important for raising the profile of the program amongst community members and enhancing engagement. Secondly, strong community links and support was necessary to assist the program to mobilize extra-curricular activities. The involvement of the local leaders, community resource persons and the peer-leaders at each stage was crucial, whilst also challenging to undertake in a way that did not compromise the program fidelity. Thirdly, the program content needed to be relevant to the participants. Finally, the peer-leaders required on-going support to enhance confidence and motivation to run the program. The use of lay peer-leaders over health professionals adds strength to this study as it overcomes the need to rely on resource-intensive health professionals. The resulting program, K-DPP, was built around a core of peer-led, community-based small group sessions supported by expert-led education sessions and extracurricular activities.

## Conclusion

The development of K-DPP demonstrates the feasibility of adapting evidence-informed prevention programs to LMIC settings such as India. The phased approach to program development and adaptation from HIC employed in this study could be utilized for cultural translation of any program. The process systematically explored the local context and interlinked it with evidence-based behavior change techniques tested elsewhere. The knowledge generated through the process of translation of K-DPP will benefit policy makers, action implementers, service providers, and eventually wider populations in India and similar countries. Lessons learnt from the implementation of K-DPP would also have applicability to other rapidly developing LMICs in the Asia, Pacific and African regions where there is an urgent need of such interventions to control the growing numbers of cases with chronic diseases.

## Abbreviations

ASHAs: Accredited social health activists
BCT: Behavior change technique (BCT) Taxonomy v1
DPES 1: Diabetes prevention education session 1
DPES 2: Diabetes prevention education session 2
GGT DPP: Greater green triangle diabetes prevention project
HIC: High income country
K-DPP: Kerala diabetes prevention program
LMIC: Low- and middle income country
NCD: Non-communicable disease
OGTT: Oral glucose tolerance test
SCTIMST: Sree Chitra Tirunal Institute for Medical Science and Technology
T2DM: Type 2 diabetes mellitus
US DPP: United States Diabetes Prevention Program
